# A continuous improvement agenda for WHO’s normative and standard-setting functions

**DOI:** 10.2471/BLT.24.292540

**Published:** 2024-10-01

**Authors:** Lisa Askie, Kidist Bartolomeos, Jeremy Farrar, Mubashar Sheikh

**Affiliations:** a Science Division, World Health Organization, Avenue Appia 20, 1211 Geneva 27, Switzerland.

A foundational element of the World Health Organization’s (WHO) mandate^1^ is to provide its Member States with scientific advice, normative leadership and technical support. This role is realized by developing evidence-based guidelines and other normative products that guide Member States in their public health decisions and actions, and by assisting them to implement these recommendations in their contexts.^2^ Member States reaffirmed this role by approving the Organization’s General Programme of Work for 2025–2028, which has a specific focus on country-level impact.^3^ Despite this core function, at critical past junctures^4^ and in times of global crises and conflict, WHO’s normative role and relevance have been questioned.^5^

In the 2023 call for papers for this theme issue of the *Bulletin of the World Health Organization*,^6^ we asked for contributions that would highlight successes and failures with respect to WHO’s normative function to understand where and how WHO normative guidance has been impactful, lessons learnt for future improvement, and what changes might be needed to improve WHO’s standard-setting role.

The papers included showcase a range of examples and experiences demonstrating the ongoing value of WHO’s normative leadership. Several articles highlight the considerable effort required to adapt globally produced WHO guidance to make it fit-for-purpose for those who use it locally.^7^^–^^10^ Other articles suggest ways WHO’s normative guidance might be improved in terms of its relevance, currency, dissemination, reach and impact. The Organization’s overall mark on this core role indicates there is still room for improvement.

Important and pioneering strides have been made, including through the *WHO* *Model list of essential medicines*.^11^ Although more can be done to maximize its impact at country level,^12^ the model list has led to major advances in access to affordable essential medicines in many countries. Progress has also been made with respect to community engagement in guideline development, as in the consolidated guideline on sexual and reproductive rights for women living with human immunodeficiency virus.^13^ Similarly, the WHO-INTEGRATE framework^14^ was designed to ensure that human rights, equity, as well as social and environmental impact are more meaningfully and explicitly considered when developing normative guidelines. Powerful examples exist of where WHO’s mandate in providing global, cross-border normative guidance and leadership in otherwise regulation-free zones remains critical, such as for medical products of human origin.^15^

Interestingly, however, many papers show how WHO’s normative guidance, as currently produced, often requires significant adaptation, scale-up or other modifications before it is used in national settings. For example, many papers spanning an array of health topics point to the need for improvements in the integration of local insights, contextualization and adaptation of WHO guidelines for use in different health systems such as in countries and territories of the Caribbean,^7^ Estonia^8^ and Indonesia.^9^ In many settings, including Malawi, Nigeria and South Africa,^10^ greater local capacity and expertise are needed to develop context-specific evidence, including local health economic analyses, to ensure global guidelines are relevant.

Notably, few articles reported on research that measured or demonstrated the impact of normative guidance for its intended users. One paper that addressed this issue reports on a natural experiment assessing the uptake of voluntary versus mandatory food nutrition labels in Australia.^16^ Another documents the ways in which systematic monitoring and supported supervision in the WHO Reaching Every District (RED) approach led to increased immunization coverage in the Solomon Islands.^17^ Better methods for assessing the impact of WHO’s normative work at country level are clearly needed.^18^

Global changes will continue in the economic, demographic, environmental, technological, cultural and scientific landscape. With nearly all sustainable development goals off track halfway to 2030, progress has eroded or stagnated across many areas, including health. WHO must become more efficient, relevant and responsive to emerging global and national public health challenges. More specifically, WHO seeks to bring science further to the forefront of all its work to achieve a higher impact on people’s health. To do so, WHO could develop new ways of producing, disseminating, updating and embedding the Organization’s normative guidance into point-of-care systems, thereby putting evidence at the centre of everyday life.^19^^,^^20^ The papers in this issue demonstrate that WHO’s normative leadership role in achieving health for all remains important and a core organizational mandate. We need better and more sustainable ways to support, streamline and measure the impact of WHO’s normative guidance to ensure it remains trusted and timely, useful and used, and accessible and equitable for all.
